# Microproteins: Overlooked regulators of physiology and disease

**DOI:** 10.1016/j.isci.2023.106781

**Published:** 2023-04-29

**Authors:** Keira R. Hassel, Omar Brito-Estrada, Catherine A. Makarewich

**Affiliations:** 1 The Heart Institute, Division of Molecular Cardiovascular Biology, Cincinnati Children’s Hospital Medical Center, Cincinnati, OH 45229, USA; 2 University of Cincinnati College of Medicine, Cincinnati, OH 45229, USA; 3 Department of Pediatrics, University of Cincinnati College of Medicine, Cincinnati, OH 45229, USA

**Keywords:** Biological sciences, Biochemistry, Molecular biology, Cell biology

## Abstract

Ongoing efforts to generate a complete and accurate annotation of the genome have revealed a significant blind spot for small proteins (<100 amino acids) originating from short open reading frames (sORFs). The recent discovery of numerous sORF-encoded proteins, termed microproteins, that play diverse roles in critical cellular processes has ignited the field of microprotein biology. Large-scale efforts are currently underway to identify sORF-encoded microproteins in diverse cell-types and tissues and specialized methods and tools have been developed to aid in their discovery, validation, and functional characterization. Microproteins that have been identified thus far play important roles in fundamental processes including ion transport, oxidative phosphorylation, and stress signaling. In this review, we discuss the optimized tools available for microprotein discovery and validation, summarize the biological functions of numerous microproteins, outline the promise for developing microproteins as therapeutic targets, and look forward to the future of the field of microprotein biology.

## Graphical abstract

**Figure:**
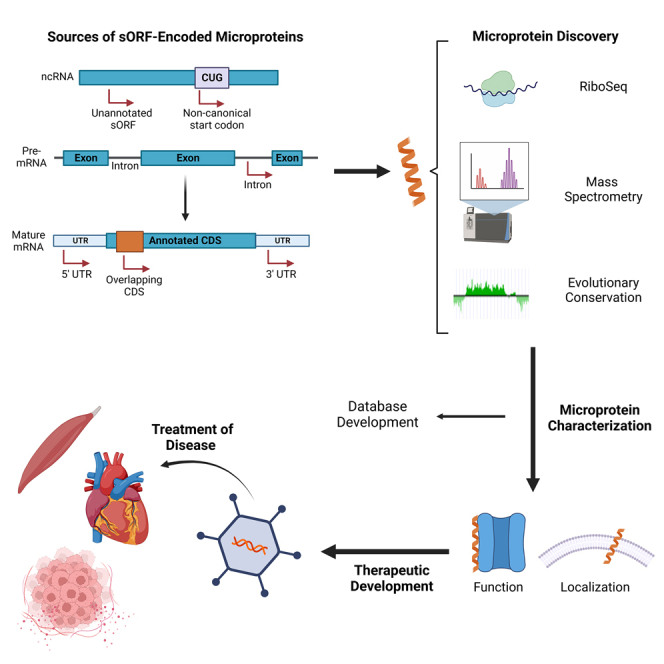

## Introduction

Recent studies have shown that the mammalian genome harbors hundreds of previously unannotated short open reading frames (sORFs) with the potential to code for functional small proteins called microproteins.^1^^,^^2^^,^^3^^,^^4^^,^^5^ Microproteins are typically less than 100 amino acids (AAs) in length, and until now, they have evaded detection because traditional genome annotation methods relied on stringent rules to distinguish protein coding RNAs versus non-coding RNAs (ncRNAs) to minimize the discovery of false positives including a minimum ORF length of 300 base pairs (bps). This ad hoc 100-codon threshold was initially selected based on the calculated probability that ORFs over 300 bps are significantly more likely to encode stable proteins.^6^ sORF-encoded microproteins have emerged as important new players in cellular biology and physiology, and they continue to be identified at high rates.^1^^,^^2^^,^^3^^,^^4^^,^^5^

Of course, small proteins (i.e., peptides) have long been recognized as key players in biology such as the essential hormone peptide insulin,^7^ and the neuropeptides substance P^8^ and neuropeptide Y.^9^ However, these peptides are fundamentally different from microproteins in that they are synthesized as larger precursor molecules (i.e., preproproteins) and are post-translationally processed and cleaved by proteases to generate their active peptide product.^10^ At the genomic and mRNA level, microproteins and preproproteins share many features. Their genes can be comprised of single or multiple exons, and their mRNAs are post-transcriptionally processed prior to translation (capped at the 5′ end, spliced, and 3′ polyadenylated). However, microproteins are translated directly from their mRNA as mature, functional proteins and are not typically products of post-translational cleavage.

Microproteins participate in diverse biological processes and are under investigation as possible therapeutic targets for diseases such as heart failure, obesity, and cancer. A large number of identified microproteins function as allosteric regulators of larger proteins and modify or fine-tune their activities,^11^^,^^12^^,^^13^^,^^14^ while others have been shown to work independently as signaling molecules or effector proteins.^15^^,^^16^^,^^17^ Interestingly, a large portion of microproteins functionally characterized thus far contain conserved transmembrane domains and localize to the plasma membrane or membranes of subcellular organelles where they exert their functions.^12^^,^^13^^,^^14^^,^^18^^,^^19^^,^^20^^,^^21^^,^^22^^,^^23^ While it is possible that microproteins are enriched in membrane domains, another possibility is that current microprotein identification methods are inherently biased for the identification of membrane microproteins due to the presence of their highly conserved transmembrane domains, which can be readily detected using domain prediction tools.^24^^,^^25^^,^^26^ Therefore, it is important to continue to optimize and implement new tools and methods to identify microproteins independent of their domain structure and subcellular localization.

## Tools and methods for microprotein discovery

Over the past decade, dedicated efforts have been made to develop tools and methods to identify and validate sORF-encoded microproteins. Each of these approaches has their own strengths and limitations, therefore combinatorial approaches are now being applied with improved outcomes. The specific tools and challenges associated with microprotein identification have been extensively discussed in several excellent recent reviews.^27^^,^^28^^,^^29^ Thus, here we will introduce only a small subset of the methods that have been developed to overcome these obstacles and successfully applied to identify microproteins.

### Ribosome profiling

**Figure 1:**
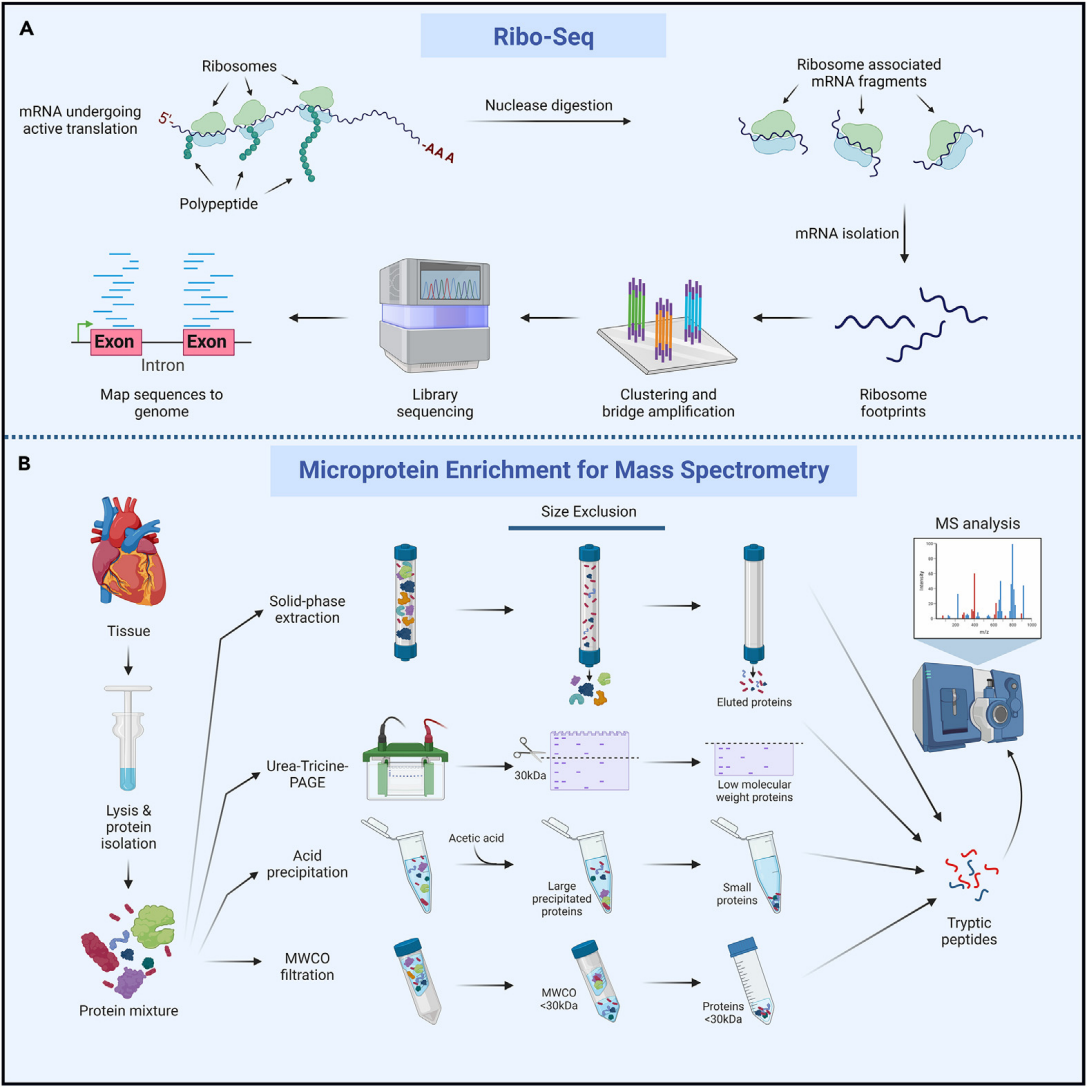
Methods for the identification of microproteins (A) Ribosome profiling (Ribo-seq) methods provide a means to query the translational landscape of cells and tissues. Ribosomes are isolated and samples are treated with nucleases to digest unprotected regions of mRNA. Resultant ribosome-protected mRNA fragments are then sequenced and mapped back to the genome. (B) Microprotein enrichment strategies for mass spectrometry (MS)-based identification methods. These include solid-phase extraction, urea-tricine polyacrylamide gel electrophoresis (PAGE), acid precipitation, and molecular weight cut-off filtration (MWCO, shown here with a 30 kDa filter).

Ribosome profiling, or Ribo-seq (ribosome sequencing), is a powerful method that generates an unbiased genome-wide snapshot of active translation which can be utilized to identify sORFs with protein-coding potential.^30^ Ribosome “footprints”, typically ∼28–30 nucleotides in length, are generated from ribosome-bound mRNA that is protected from nuclease digestion (Figure 1A). These footprints are sequenced and mapped to the genome to identify regions undergoing active translation including putative sORF-encoded microproteins. Ribo-seq can capture instantaneous translation events in response to signals or stimuli, helping to identify sORFs that may only be translated under specific conditions.^31^ While Ribo-seq provides high sensitivity to map ORFs in the genome, these results must be experimentally validated as it has been demonstrated that ribosomes can associate with mRNAs without undergoing active translation.^32^ Despite this limitation, Ribo-seq is a robust tool that can be applied to identify sORFs, and there are ongoing efforts to increase the sensitivity of Ribo-seq to the single cell level to capture cell-specific transcripts,^33^ which may aid in the identification of cell-type specific microproteins in the future.

### Microprotein enrichment for MS

Mass spectrometry (MS) is the gold standard for protein identification and quantification. For MS analysis, proteins are first isolated from cells or tissue extracts and then processed by a series of steps (reduction, alkylation, trypsin digestion, de-salting, and peptide enrichment) that provide tryptic peptides for liquid chromatography with tandem MS (LC-MS/MS).^34^ However, while MS is a powerful method used for protein identification, there are several factors that limit the ability of standard MS approaches to detect microproteins. First, many small proteins are often lost in the sample preparation steps leading up to MS.^27^ Second, MS is inherently biased for large proteins that can produce hundreds of unique tryptic peptides, while microproteins often yield only a single peptide after protease digestion making their detection more difficult.^34^^,^^35^ Third, many microprotein sequences are not contained in reference protein databases since they are not yet annotated, therefore unique peptide fragments often go unmapped.^29^

To overcome some of these challenges, new microprotein enrichment techniques are being used to exclude larger proteins and enrich for microproteins prior to protease digestion to facilitate their identification (Figure 1B). Some of the most commonly used methods for microprotein enrichment include solid-phase extraction, urea-tricine polyacrylamide gel electrophoresis (PAGE), acid precipitation, and molecular weight cut-off (MWCO) filtration.^34^^,^^36^ These methods have resulted in the identification of several microproteins,^37^^,^^38^^,^^39^^,^^40^ and when combined with sequencing-based or bioinformatic approaches, they may be the most robust approach for high confidence microprotein discovery and identification.^34^ This was recently highlighted with the implementation of comprehensive proteogenomic approaches combining peptidomics and RNA sequencing to filter out known peptides and exclusively identify new microproteins.^41^^,^^42^ Lastly, it may be useful to implement data independent acquisition (DIA) during MS analysis. DIA analyzes the full spectrum detection range and can identify lower abundant peptides, such as microproteins, that are typically lost in traditional data dependent acquisition (DDA).^29^^,^^43^

### Proximity ligation-based assays

BioID proximity ligation methods have recently been used to identify microproteins and elucidate their interactomes.^44^^,^^45^^,^^46^ These techniques make use of promiscuous biotin ligase enzymes, such as MicroID (TurboID)^44^ or APEX,^45^ which are fused to microproteins of interest or bait proteins and covalently biotinylate proteins in close proximity (1-10 nm) upon treatment with biotin (MicroID) or biotin phenol with hydrogen peroxide (APEX). Biotinylated proteins are subsequently purified using biotin-streptavidin-based systems and identified by MS analysis. For example, the APEX system was recently used to identify interacting partners of the uncharacterized microprotein C11orf98.^45^ Additionally, a recent study elegantly applied the MicroID system to identify numerous microproteins and alternative proteins (alt-proteins) in subnuclear compartments both *in vitro* and *in vivo*^44^ highlighting the ability of this method to be implemented in the discovery of microproteins when coupled with MS. While these proximity ligation assays have enabled the discovery of microproteins, it is important to note that these systems rely on treatment with biotin or hydrogen peroxide which are found endogenously and may yield false positives, thus necessitating experimental validation.^44^

### Publicly available bioinformatic tools and databases

Numerous bioinformatic tools and databases have been developed for the identification of microproteins and a subset of these tools is featured in Table 1. Additionally, a recent review highlighted many of the tools and databases that are publicly available^29^ including SmProt^47^ and sORFs.org,^48^ which contain thousands of predicted sORFs with protein coding potential. However, concerns have been raised regarding the stark difference between both databases, though both used many of the same datasets for construction.^5^ This is presumably because each applied different criteria for classifying putative coding ORFs, further highlighting the need for more cohesive approaches for classifying sORFs. Collaborative efforts to standardize the annotation of translated sORFs are currently underway and will facilitate high confidence microprotein identification in the future.^49^ Still, while there are a growing number of bioinformatic tools available to identify sORF-encoded microproteins, there is still a need to continue optimizing these tools and databases to specifically identify microprotein-coding potential in the genome.

**Table 1:** Publicly available bioinformatic tools and databases

Bioinformatic tools and databases	Description	Reference
PhyloCSF	PhyloCSF analyzes and scores codon substitution frequencies across multiple mammalian species and applies phylogenetic models to identify conserved patterns of AA codons.	Lin et al.^50^
sORF.org	This is a public repository for sORFs that were identified using ribosome profiling and it incorporates the sORF Blast tool that looks at sequence similarities between species as well as MS data with their predictions.	Olexiouk et al.^48^
MiPepid	The algorithm for MicroPeptide identification (MiPepid) uses machine learning from microprotein datasets rather than canonical proteins to identify microproteins from DNA sequences.	Zhu et al.^51^
smORFunction	This tool is designed to predict the function and localization of sORF-encoded microproteins from nucleotide or AA sequences, or input human genome coordinates. This tool uses a nearest neighbor algorithm approach to predict their function.	Ji et al.^52^
uPEPperoni	This is a tool that uses sequence conservation techniques to detect ORFs in the 5′ UTR regions of mRNAs. uPEPperoni incorporates substitution frequency scores and heatmaps that allow for visualization of conserved regions of an mRNA molecule.	Skarshewski et al.^53^
OPENProt	This MS-based proteomics tool uses polycistronic models for eukaryotes that allows the detection of multiple ORFs	Brunet et al.^54^
SPADA	Small Peptide Alignment Discovery Application (SPADA) uses homology to predict plant microprotein-coding genes in a region of interest by analyzing its sequence and integrating information from other gene families.	Zhou et al.^55^

Here, we will highlight one such tool, PhyloCSF, which is a conservation-based algorithm that has been successfully used to identify numerous microproteins.^11^^,^^12^^,^^13^^,^^14^^,^^50^^,^^56^^,^^57^^,^^58^^,^^59^ A hallmark feature of protein coding ORFs is their high degree of sequence conservation at the AA (codon) level, which differs from ncRNAs that are typically not highly conserved. These conservation signatures can be leveraged to identify sORF-encoded microproteins using PhyloCSF, which applies a phylogenetic sequence conservation algorithm to analyze and score codon substitution frequencies across >50 mammalian species.^50^ PhyloCSF positively scores synonymous codon substitutions (those that encode the same AA) or AAs with similar properties (polarity, charge, hydrophilicity, etc.), while nonsynonymous or missense substitutions are scored negatively. Importantly, PhyloCSF can classify very short portions of coding sequences in isolation from the full sequence, which is necessary when considering individual exons and this inherent property of the program can be leveraged to help identify sORFs with microprotein-coding potential.^49^^,^^50^ PhyloCSF is user-friendly and can be easily accessed and used via the UCSC Genome Browser.^60^

### Microprotein characterization and validation

It is important to note that while bioinformatic and computational approaches can be readily applied to assess microprotein-coding potential for genomic regions of interest, such predictions do not guarantee that identified coding ORFs will be translated into functional protein products. Therefore, identified microproteins must be validated by experimental approaches such as raising a sequence-specific antibody to detect the microprotein of interest. A caveat to antibody-based validation is that due to the inherent small size of microproteins, there are limited options for target antigen design for antibody production, and available sequences may not be suitable to generate strong antigenicity. Additionally, since many microproteins contain transmembrane domains or are associated with larger proteins, the antibody-binding site may not be readily available under native conditions for recognition using methods such as immunoprecipitation and immunocytochemistry. Alternatively, the CRISPR/Cas9 genome editing system can be used to insert a coding sequence for an epitope tag (e.g., HA, FLAG, myc) in-frame with the microprotein of interest within its endogenous locus via homology-directed repair (HDR).^61^ When using CRISPR/Cas9 HDR knock-in methods to generate epitope-tagged fusion proteins, it is ideal to screen knock-in clones with the tag inserted into the N- and C-terminus of the sORF of interest because the properties of the tags themselves may influence microprotein localization and function.^61^ Many of these methods have been successfully implemented to elucidate the microproteins described in detail below.

## The functions of microproteins in distinct subcellular domains

sORF-encoded microproteins play diverse functions in cellular physiology that can be attributed in part to their localization to specific subcellular structures and organelles including the plasma membrane, sarco/endoplasmic reticulum (S/ER), endo/lysosome, mitochondria, cytoplasm, and nucleus. When characterizing new microproteins, deciphering their subcellular localization is an important step toward determining potential interacting partners and understanding their function. Here, we will introduce numerous newly discovered microproteins and discuss the critical biological functions they perform in discrete subcellular domains (summarized in Table 2).

**Table 2:** Summary of the microproteins discussed in this review with UniProt identifiers (IDs) and amino acid (AA) lengths

Microprotein	Function	Human gene	Human AA length	Human UniProt ID	Mouse gene	Mouse AA length	Mouse UniProt ID
ALN	Negative regulator of SERCA	*C4orf3*	66	Q8WVX3	*1810037I17Rik*	65	Q99M08
APPLE	Regulator of translation initiation through promotion of elF4A initiation complex assembly	*ASH1L-AS1*	90	NR	NR	NR	NR
ASAP	Positive regulator of mitochondrial ATP production through interaction with ATP synthase	*LINC00467*	94	NR	NR	NR	NR
BRAWNIN	Electron transport chain complex III assembly factor	*UQCC6*	71	Q69YU5	*Uqcc6*	67	Q8BTC1
CASIMO1	Regulator of cytoskeletal organization, cell migration and proliferation	*SMIM22*	83	K7EJ46	*Smim22*	86	V9GXA9
CIP2A-BP	Negative regulator of PI3K/AKT/NFkB pathway through interaction with CIP2A	*LINC00665*	52	NR	NR	NR	NR
CYREN-1/MRI-1	Regulator of non-homologous end-joining	*CYREN*	157	Q9BWK5-1	*Cyren*	157	Q8BHZ5
CYREN-2/MRI-2	Regulator of non-homologous end-joining	*CYREN*	69	Q9BWK5-4	NR	NR	NR
DWORF	Postive regulator of SERCA2a in heart and slow-twitch skeletal muscle fibers	*STRIT1/DWORF*	35	P0DN84	*Strit1/Dworf*	34	P0DN83
ELN	Negative regulator of SERCA3	*SMIM6*	62	P0DI80	*Smim6*	56	Q3U0I6
Humanin	Anti-apoptotic activity	*MT-RNR2*	24	Q8IVG9	*Gm20594*	37	J3QJY3
MIAC	Negative regulator of actin cytoskeleton through regulation of SEPT2/ITGB4 signaling	*RP11-469H8.6/AQP5-AS1*	51	NR	NR	NR	NR
NR miPEP133	Regulator of p53 transcription and mitochondrial HSPA9 activity	*MIR34A*	133	NR	*Mir34a*	NR	NR
MLN	Negative regulator of SERCA1	*MRLN*	46	P0DMT0	*Mrln*	46	Q9CV60
MOTS-c	Regulator of insulin sensitivity and glucose metabolism	*MT-RNR1*	16	A0A0C5B5G6	*mt-Rnr1*	11	NR
MP31	Negative regulator of lactate-pyruvate conversion through interaction with mitochondrial lactate dehydrogenase	*ENSG00000289051* 5′ UTR of *PTEN* (uORF)	31	C0HLV8	*ENSMUSG00000121574* 5′ UTR of *Pten* (uORF)	35	C0HLV9
Mtlbn	Regulator of electron transport chain complex III assembly and function	*STMP1/C7orf73*	47	E0CX11	*Stmp1/1810058I24Rik*	47	P0DP99
Mtln	Regulator of fatty acid oxidation, lipid metabolism and respiratory chain activity	*MTLN*	56	Q8NCU8	*Mtln*	56	Q8BT35
Myomerger/Myomixer	Regulator of myoblast fusion	*MYMX*	84	A0A1B0GTQ4	*Mymx*	84	Q2Q5T5
NEMEP	Regulator of glucose transport and mesendoderm development	*SMIM43*	63	Q4W5P6	*Smim43*	63	A0A286YD83
NoBody	Regulator of mRNA stability through P-body interaction	*NBDY*	68	A0A0U1RRE5	*Nbdy*	68	A0A0N4SUI7
PIGBOS	Regulator of the ER stress response and UPR	*PIGBOS1*	54	A0A0B4J2F0	*Pigbos1*	57	A0A5F8MPY0
PLM/FXYD1	Negative regulator of NKA and NCX	*FXYD1*	92	O00168	*Fxyd1*	92	Q9Z239
PLN	Negative regulator of SERCA2a	*PLN*	52	P26678	*Pln*	52	P61014
pTINCR	Regulator of CDC42 SUMOylation and activation	*TINCR*	87	A0A1B0GVN0	*Tincr*	87	A0A1B0GRQ3
pTUNAR	Postive regulator of SERCA2 activity during neural differentiation	*TUNAR*	48	A0A1B0GTB2	*Tunar*	48	A0A1B0GQX2
SMIM4	Electron transport chain complex III assembly factor	*UQCC5*	70	Q8WVI0	*Uqcc5*	80	Q8C1Q6
SPAR	Regulator of v-ATPase H+ pump and inhibitor of mTORC1	*SPAAR*	90	A0A1B0GVQ0	*Spaar*	75	A0A1B0GSZ0
Stannin/Hemotin	Regulator of endosomal maturation and phagocytic processing in macrophages	*SNN*	88	O75324	*Snn*	88	P61807
UQCC3	Electron transport chain complex III assembly factor	*UQCC3*	93	Q6UW78	*Uqcc3*	89	Q8K2T4
UQCC4	Electron transport chain complex III assembly factor	*UQCC4*	132	Q4G0I0	*Uqcc4*	136	Q6RUT7

NR, Not Reported.

### Plasma membrane

**Figure 2:**
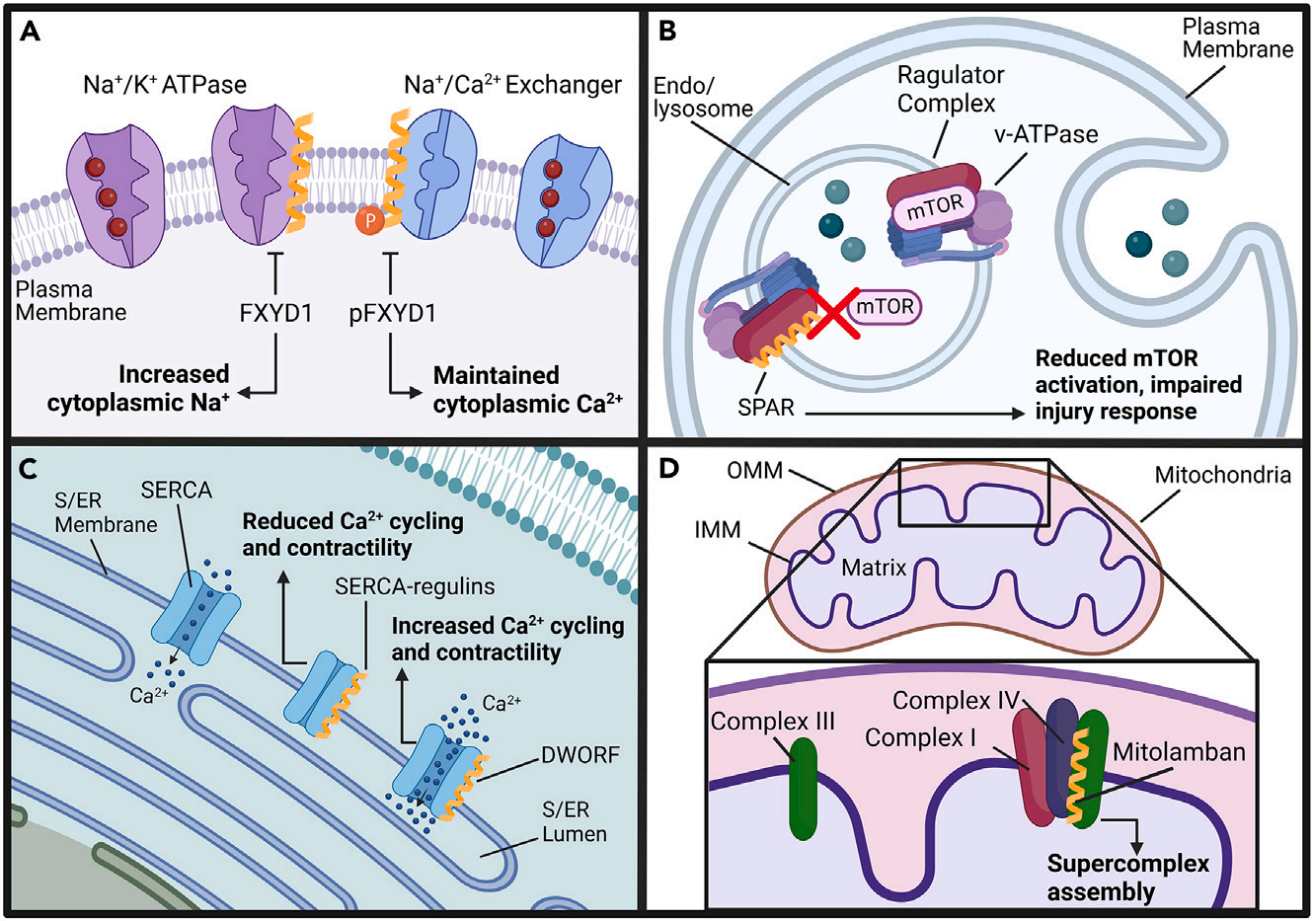
Membrane microproteins have diverse functions throughout the cell (A) FXYD1 (PLM) in the plasma membrane interacts with the Na^+^/K^+^ ATPase and inhibits its function when dephosphorylated, increasing cytoplasmic Na^+^. FXYD1 inhibits the cardiac Na^+^/Ca^2+^ exchanger when phosphorylated to prevent cytoplasmic Ca^2+^ export and maintain contractility in cardiomyocytes. (B) SPAR localizes to the endo/lysosomal membrane and inhibits incorporation of mTORC1 into the Ragulator complex, decreasing activation of mTORC1 and impairing injury response. (C) The SERCA-regulins interact with SERCA in the S/ER membrane to decrease SERCA Ca^2+^ affinity, Ca^2+^ cycling, and contractility in myocytes. In the heart, DWORF interacts with SERCA and enhances its Ca^2+^ transport activity to increase Ca^2+^ cycling and cardiac contractility. Ca^2+^, calcium; S/ER, sarco/endoplasmic reticulum; SERCA, S/ER calcium ATPase. (D) Mitolamban localizes to the IMM and interacts with complex III to assist with mitochondrial respiratory supercomplex assembly and function. OMM, outer mitochondrial membrane; IMM, inner mitochondrial membrane.

In addition to partitioning individual cells, the plasma membrane is responsible for mediating functions such as ion exchange, cell fusion, and signal transduction. Many of these processes are controlled by large protein complexes that are subjected to regulation by microproteins. For example, the Phe-Xaa-Tyr-Asp (FXYD) family (FXYD1-7) are type I single-pass transmembrane microproteins that regulate ion transport at the plasma membrane. FXYD proteins contain an invariant PFXYD motif in their extracellular N-terminal domain that enables them to serve as auxiliary subunits of the sodium (Na^+^)/potassium (K^+^) ATPase (NKA).^18^^,^^62^ NKA is a plasma membrane enzyme that generates an electrochemical gradient for Na^+^ and K^+^ across the membrane by exporting 3 Na^+^ ions and importing 2 K^+^ ions for every ATP molecule hydrolyzed. This ubiquitous membrane transporter is essential for maintaining cell volume, pH, nutrients, and ion gradients that supply energy for secondary membrane transport. FXYD proteins were recognized for several decades as small proteins (61-95-AA, except FXYD5 at 178-AA) that could affect ion transport when overexpressed, though their function and interactions remained unknown until more recently.^18^ FXYD2 (γ-subunit of NKA) was the first member of the family defined to be a regulator of NKA. Expressed primarily in the kidney,^63^ FXYD2 regulates NKA ion affinity depending on membrane potential. In addition, human mutations in FXYD2 have been linked to primary hypomagnesemia,^64^ representing one of the few known instances of a pathology-causing microprotein. The other members of the FXYD family are expressed in a tissue-specific manner, allowing for precise regulation of NKA throughout the body. The cardiac-enriched FXYD1, also called phospholemman (PLM) is depicted in Figure 2A.

PLM is one of the best examples of the dynamic actions of microproteins and their ability to fine-tune cellular physiology. Originally described to interact with and regulate NKA, PLM was subsequently found to also be an endogenous regulator of the Na^+^/calcium (Ca^2+^) exchanger (NCX),^19^ which led to extensive investigation into its role in regulating skeletal and cardiac muscle contractility.^65^ PLM activity, and therefore NKA and NCX function, is regulated by the phosphorylation state of PLM on cytoplasmic serine residues 63 and 68.^66^^,^^67^ PLM is phosphorylated by protein kinase A (PKA) and protein kinase C (PKC) during stress and catecholamine release, which induces its dissociation from NKA and relief of inhibition. In contrast, phosphorylated PLM promotes inhibition of NCX via interaction with the intracellular loop.^66^^,^^68^ The elegant nature of PLM activity is evident when viewed at the level of the cardiomyocyte: during stress responses, PLM is phosphorylated which relieves inhibition of NKA and decreases intracellular Na^+^.^19^ The relative loss of intracellular Na^+^ activates NCX, which in-turn reduces cytoplasmic Ca^2+^.^19^ However, because phosphorylated PLM inhibits NCX activity, intracellular Ca^2+^ loss is limited. These coordinated efforts simultaneously lower the risk for arrythmia while preserving contractility^19^ (Figure 2A).

Plasma membrane microproteins function beyond ion exchange to affect other critical processes such as fusion and cell signaling during development. For example, the 63-AA microprotein NEMEP (Nodal Enhanced MEsendoderm Peptide) is a transmembrane microprotein that helps regulate early mesendoderm development.^69^ Discovered as a putative ncRNA target of Nodal, NEMEP localizes to the plasma membrane and interacts with the glucose transporters GLUT1 and GLUT3 to directly enhance glucose transport into cells. NEMEP loss-of-function studies demonstrate impaired mesodermal differentiation and glucose uptake *in vitro* and mesodermal developmental defects *in vivo*.^69^

Similarly, the 84-AA microprotein myomerger,^70^ also called myomixer^71^ and minion,^72^ is important for myoblast fusion during muscle formation. It is now known that two myoblast proteins, myomerger and the 221-AA protein myomaker, regulate fusion in a stepwise manner, requiring first myomaker on both cells and then myomerger on only one cell to complete the process.^73^ Myomerger-null mice do not undergo myoblast fusion which leads to neonatal lethality,^70^^,^^71^^,^^72^ marking myomerger as a microprotein that is essential for muscle formation and viability.

### Endo/lysosomes

Endocytosis is a critical fundamental cellular process that utilizes membrane-bound vesicles to partition functions such as signal transduction, energy sensing, autophagy, and exogenous protein digestion within the endo/lysosome.^74^ Microproteins contribute to several distinct aspects of these processes, such as the 83-AA cancer associated small integral membrane open reading frame 1 (CASIMO1). Using a microarray-based approach, CASIMO1 was discovered as a sORF-encoded microprotein upregulated in breast cancer samples.^75^ Functional characterization revealed co-localization of CASIMO1 with the late endosomal marker LAMP1, leading to the discovery of CASIMO1’s role in actin cytoskeletal organization, cell migration, and proliferation, which are key features of cancer progression and prognosis.

SPAR is another endo/lysosomal-associated microprotein that was discovered through MS-based proteomic screening of ncRNAs.^76^^,^^77^ The ncRNA LINC00961 was revealed as a candidate microprotein and validated to code for a 90-AA protein that localizes to the late endo/lysosome and interacts with the membrane-associated v-ATPase hydrogen (H^+^) pump. Results further showed SPAR as an inhibitor of mTORC1, a key player in cell growth and proliferation in response to skeletal muscle injury^78^ (Figure 2B). SPAR is now recognized to have important implications for skeletal muscle regeneration and continues to be investigated as a regulator of tissue injury response.

Several human microproteins have been identified to have *Drosophila melanogaster* homologs, including the 88-AA hemotin^79^ which functions at the late endosome. Hemotin is a necessary component of the *Drosophila* immune system via regulation of phagocytosis in macrophage-like cells, a function which is shared with its human homolog, stannin. Stannin promotes endosomal maturation and phagocytic processing in macrophages,^79^ confirming the evolutionarily conserved function of hemotin/stannin in organismal immunity.

### Sarco/endoplasmic reticulum

The S/ER regulates cellular homeostasis on multiple fronts including protein synthesis, protein folding, and storage of intracellular Ca^2+^. Ca^2+^ is a ubiquitous second messenger that participates in numerous cell-signaling events upon its release from the S/ER into the cytosol.^80^ Ca^2+^ is actively transported back into the S/ER via the S/ER Ca^2+^ ATPase (SERCA) to restore basal cytoplasmic Ca^2+^ levels. SERCA enzymes are large (994–1042 AA) P-type ATPase pumps that are embedded in the S/ER membrane and their activity is potently regulated by a family of microproteins termed the “SERCA-regulins” including PLN (52-AA), ALN (65-AA), ELN (56-AA), MLN (46-AA), and SLN (31-AA)^12^^,^^20^^,^^21^^,^^22^^,^^23^ (Figure 2C). The SERCA-regulins are co-expressed with different SERCA isoforms (SERCA1a, SERCA2a, SERCA2b, and SERCA3) in a tissue-specific manner.^12^^,^^81^ These small proteins share sequence homology within their TM domain, which enables direct interaction with SERCA.^12^

The potent activity of these SERCA-regulins can be appreciated in the context of cardiomyocyte contractility. During cardiac contraction, Ca^2+^ is rapidly released from the S/ER, which induces sarcomere shortening and activates SERCA2a (the primary SERCA gene product in the heart), which uses the energy generated from ATP hydrolysis to pump Ca^2+^ back into the S/ER to induce cardiac relaxation.^82^ This process is highly regulated by the cardiomyocyte-expressed SERCA-regulins (PLN, SLN, ALN). Under normal physiological conditions, a population of SERCA2a is inhibited by PLN to maintain a “cardiac reserve” for situations where heart contractility must be rapidly increased. In response to catecholamine induced β-adrenergic signaling, PLN is phosphorylated on its cytoplasmic serine 16 residue by PKA, causing dissociation from SERCA2a. This alleviates its inhibitory effects and potently increases SERCA2a Ca^2+^ transport activity and cardiomyocyte contractility (i.e., the “fight-or-flight” response).^22^ Notably, PLN-deficient mice exhibit maximal myocardial contractile performance at baseline and completely lose the ability to respond to β-adrenergic signaling.^83^

In addition to the SERCA-regulins, dwarf open reading frame (DWORF, 34-AA) has been identified as a positive regulator of SERCA2a in cardiac and slow-skeletal muscle.^14^ The exact mechanisms that regulate DWORF’s stimulatory effects on SERCA2a are not yet fully understood, but DWORF has been shown to act in an antagonist manner to PLN by binding to the same residues on SERCA2a and actively displacing PLN from the pump to relieve its inhibitory effects, resulting in an increase in SERCA2a Ca^2+^ affinity.^14^^,^^84^ Additionally, new evidence from heterologous cell culture systems suggests that DWORF may also directly stimulate the maximal Ca^2+^ transport activity (V_max_) of SERCA2a.^85^^,^^86^ Given its potent stimulatory effects on SERCA2a and cardiomyocyte contractility, DWORF has recently emerged as a potential heart failure therapeutic.^84^^,^^87^^,^^88^ Additionally, there has been a recent report of another SERCA-interacting protein, 48-AA pTUNAR, that enhances SERCA activity during neural differentiation.^58^ Both DWORF and pTUNAR directly interact with SERCA, but their transmembrane domains lack the SERCA-inhibitory motif that is present in the SERCA-regulins,^12^^,^^58^ thus indicating that their unique transmembrane domains may mediate their distinct ability to activate SERCA.

### Mitochondria

A large number of microproteins that have been identified thus far localize to the mitochondria.^89^^,^^90^ Mitochondria are the primary organelles responsible for integrating cell signaling and survival cues to match changing metabolic and energetic demands. Several studies have identified microproteins that affect various aspects of tissue and systemic metabolism, implicating these microproteins in health and disease. One such protein is mitoregulin (Mtln),^91^^,^^92^ also known as MOXI (micropeptide regulator of β-oxidation).^13^ Previously annotated as a non-coding RNA (LINC00116), three independent groups discovered the 56-AA mitoregulin and defined its role in fatty acid oxidation, lipid metabolism, and respiratory chain activity in heart and skeletal muscle.^13^^,^^91^^,^^92^ Additional studies have described a role for mitoregulin in regulating lipolysis and mitochondrial β-oxidation in adipocytes^93^ and contributing to systemic lipid metabolism.^94^ The exact molecular mechanisms driving mitoregulin function are still under active investigation; however its involvement in lipid and fatty acid metabolism is well supported, suggesting it could be developed as a target for modifying these processes in disease.

Numerous studies have defined microproteins as essential core components, accessory subunits, and assembly factors for the electron transport chain (ETC).^56^^,^^89^^,^^91^^,^^95^^,^^96^ This is not surprising given that the ETC complexes are known to contain a disproportionately high number of small proteins. In fact, compared to the total cellular proteome where proteins under 100 AAs make up <2% of the known proteome, they constitute ∼28% of ETC proteins.^89^ Microproteins that have been recently identified that are directly involved in ETC function and supercomplex assembly (i.e., respirasomes) include BRAWNIN (71-AA)^89^ UQCC3 (93-AA),^96^ UQCC4 (132-AA),^95^ SMIM4 (70-AA),^95^^,^^97^ mitolamban,^56^ and mitoregulin^91^ (Figure 2D). Collectively, these studies highlight the important roles that microproteins play in the formation of the functional ETC and respiratory supercomplexes, processes that are currently not fully understood.

In addition to contributing to metabolism and respiration, microproteins have been implicated in organelle-organelle communication at the mitochondria. Such is the case for the 54-AA microprotein PIGBOS, which facilitates interactions between the outer mitochondrial membrane (OMM) and mitochondrial-associated ER membrane (MAM) during the stress-induced ER unfolded protein response (UPR).^57^ The UPR has been implicated in numerous neurodegenerative diseases such as Alzheimer’s, Parkinson’s, amyotrophic lateral sclerosis, and others^98^^,^^99^ and is thus an area of active investigation for targeting disease. PIGBOS increases resistance to ER-stress and apoptosis by interacting with the ER membrane protein chloride-channel CLIC-like 1 (CLCC1), suggesting a unique role for PIGBOS in inter-organelle communication and the cellular stress response.

Another mitochondrial microprotein is the anti-tumorigenic 133-AA miPEP133.^100^ miPEP133 is translated from its microRNA (miRNA) precursor, miR-34a, which is known to suppress transcription of oncogenes in multiple cancers.^101^^,^^102^^,^^103^ miPEP133 itself functions independently in an anti-tumorigenic positive-feedback loop to increase transcription of p53 and miR-34a. miPEP133 promotes anti-cancer effects by interacting with HSPA9 in the mitochondria where it inhibits interactions between HSPA9 and its binding partners. This causes changes in membrane potential, ATP production, and the cell cycle that induces apoptosis and limits tumorigenesis. miPEP133 levels correlate with favorable cancer prognosis, emphasizing the relevance of this microprotein as a prognosis factor.

### Cytoplasm

One of the earliest discovered microproteins was humanin, a 24-AA secreted protein that has been shown to be beneficial in Alzheimer’s disease (AD).^104^ Humanin is encoded by the mitochondrial genome, with its ORF found within the mitochondrial 16S rRNA gene. The beneficial effects of humanin in AD have been linked to its anti-apoptotic activity and prevention of neuronal cell death both in the cytoplasm and via cell surface receptors. Within the cytoplasm, humanin binds and stabilizes Bax, preventing downstream apoptotic signaling of the Bax/Bcl family of apoptosis-inducing proteins.^105^ Humanin also exerts cytoprotection through binding to cell surface receptors including the cytokine receptor complex CNTFR-a/WSX-1/gp130^106^ and the G-coupled protein receptor FRPL1.^107^ The interest in humanin as a promising therapeutic expands beyond AD to other neurodegenerative diseases, diabetes, and cardiovascular disease among others.^108^^,^^109^^,^^110^

The 68-AA microprotein NoBody functions within the cytoplasm to regulate mRNA stability.^111^ Previously annotated as the ncRNA LINC01420, NoBody is expressed in multiple cell lines and shows high levels of AA conservation in mammals. NoBody localizes to processing-bodies (P-bodies) in the cytosol, which regulate mRNA degradation and translation repression.^112^ P-bodies achieve partitioning though liquid-liquid phase separation, raising interesting questions about NoBody as a microprotein that functions within a cellular compartment that lacks a membrane. NoBody interacts with the mRNA decapping complex protein EDC4, leading to enhanced mRNA decapping, decreased mRNA stability, and increased mRNA degradation. Therefore, there is an inverse correlation between NoBody levels and the number of P-bodies, leading to effects on mRNA stability and overall gene expression.

### Nucleus

The nucleus is the home of many highly regulated processes, and therefore, it is not surprising that numerous microproteins localize to this site of complex cellular function. Two such examples include the splice isoforms MRI-1 (modulators of retrovirus infection homolog 1) and MRI-2 which modulate distinct pathways of DNA repair. MRI-1 was first described as a 157-AA modulator of retroviral infection,^113^ though later studies discovered its role in regulating DNA repair, leading to its renaming to CYREN (Cell cYcle REgulator of NHEJ).^114^ CYREN suppresses the error-prone and possibly genotoxic non-homologous end-joining (NHEJ) DNA repair pathway in favor of the more accurate homologous recombination (HR). CYREN achieves this by inhibiting the NHEJ proteins Ku70 and Ku80, allowing HR to outcompete NHEJ in the S/G2 cell cycle phases. In contrast, its splice isoform MRI-2 codes for a 69-AA microprotein that localizes to the nucleus and associates with the Ku-DNA NHEJ complex.^115^ In instances of DNA double-strand breaks, MRI-2 increases the rate of NHEJ to promote DNA repair and prevent apoptosis. CYREN and MRI-2 represent microprotein splice isoforms that independently regulate DNA repair and overall cell survival.

Another microprotein that localizes to the nucleus is the 87-AA pTINCR, so named due to its associated lncRNA, *TINCR*, which plays a role in epithelial differentiation.^59^^,^^116^ In mechanisms distinct from *TINCR*, pTINCR was found to promote epithelial differentiation by contributing to post-translational modification of CDC42. CDC42 is an epithelial pro-differentiation factor that has been associated with oncogenic phenotypes. However, the interaction of pTINCR with CDC42 was shown to be anti-tumorigenic and pro-differentiating, thus marking it as an anti-oncogenic factor for many epithelial cancers.

## Microproteins as therapeutic targets

As discussed in detail in this review, microproteins often function as regulators of ion channels, enzymes, or multi-protein complexes and their dysregulation can lead to disease phenotypes. The discovery of numerous microproteins that regulate critical cellular processes creates the exciting possibility of their use as therapeutic targets to treat human diseases such as heart failure and cancer. An added benefit to using microproteins over drugs or small molecule inhibitors is that such molecules are often accompanied by cytotoxicity and off-target effects while microproteins have very specific targets and may be less likely to confer cytotoxicity.

**Figure 3:**
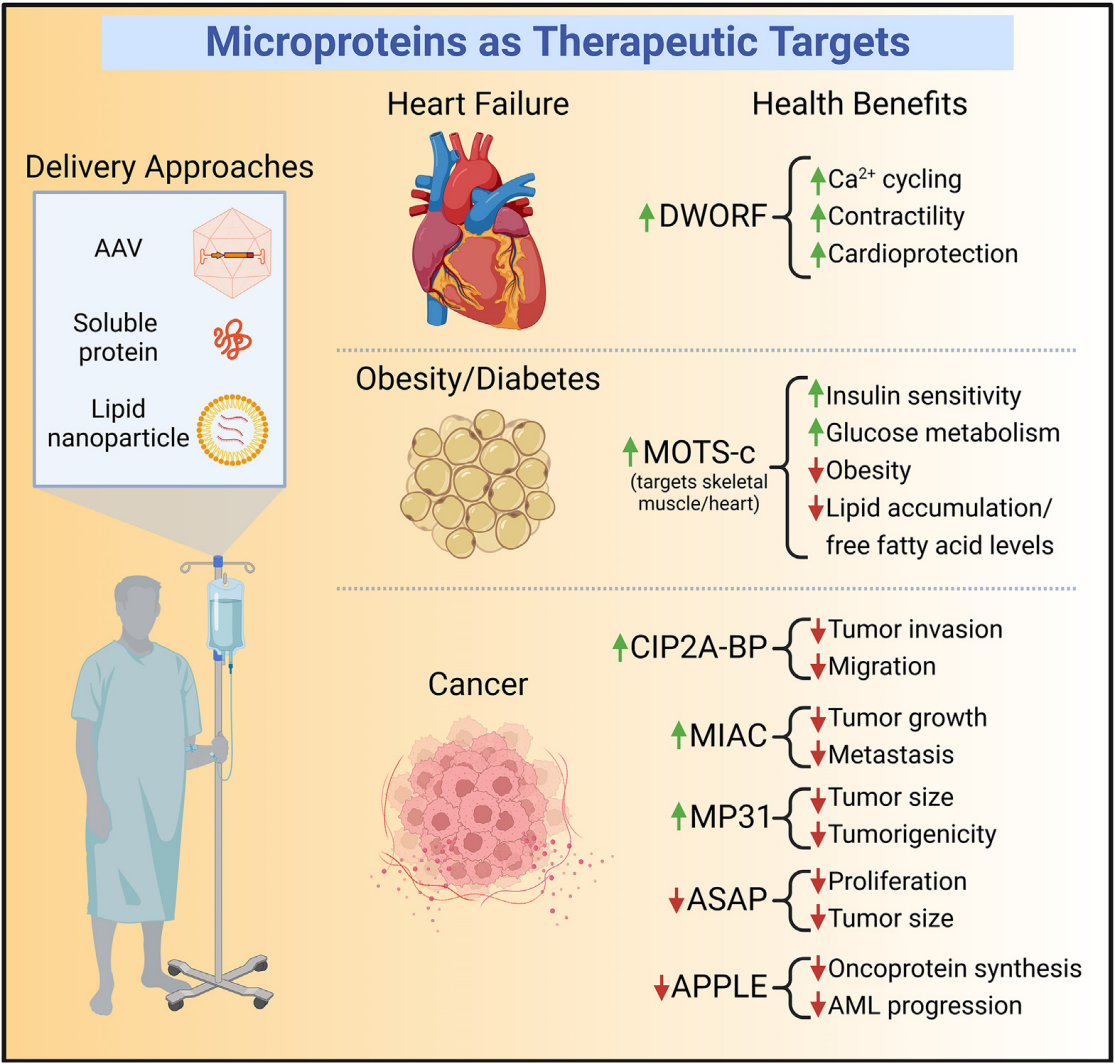
Microproteins as Therapeutic Targets Microproteins have recently gained traction as new therapeutic targets for the treatment of numerous diseases including heart failure, diabetes, obesity, and cancer. Delivery approaches for microprotein gene therapy include adeno-associated virus (AAV) methods and nanoparticle systems, which can deliver DNA, mRNA, or si/shRNA to target microproteins. MOTS-c is a soluble protein and can be directly infused into patients. AML, acute myeloid leukemia.

Microprotein gene therapy can be achieved using approaches such as adeno-associated viruses (AAVs), which can be designed to include cell-type specific promoters to drive microprotein expression and limit off-target effects.^117^ Microproteins are inherently small; therefore, they can easily meet the packaging capacity limits required for AAV.^118^ Nanoparticle delivery systems can also be used to deliver DNA, mRNA, or si/shRNAs to target microproteins^119^ and antisense locked-nucleic acid (LNA) gapmers or antisense oligonucleotides (ASOs) can also be used to modify microprotein expression levels,^120^ though these methods currently lack tissue specificity. Here we will discuss several microproteins that show promise as potential therapeutic targets for heart failure, obesity, diabetes, and cancer (Figure 3, Table 2).

### Targeting microproteins in heart failure, obesity, and diabetes

There are several microproteins that are abundantly expressed in the heart and regulate important processes such as Ca^2+^ homeostasis (the SERCA-regulins and DWORF), energy metabolism (MOXI, BRAWNIN, UQCC3, UQCC4, SMIM4, Mitolamban, MOTS-c), and ER-stress (PIGBOS). Therefore, there is great potential for these microproteins to serve as therapeutic targets to treat heart failure and obesity.

#### DWORF

Ca^2+^ dysregulation is a universal feature of heart failure and is characterized by impaired Ca^2+^ sequestration into the S/ER and reduced SERCA2a activity and expression.^121^^,^^122^^,^^123^ Therapeutic approaches that enhance the expression and/or activity of SERCA2a can restore Ca^2+^ homeostasis and ameliorate disease in animal models of heart failure and have even made it to clinical trials.^122^^,^^124^^,^^125^^,^^126^^,^^127^^,^^128^ An innovative approach to enhancing SERCA2a activity is via overexpression of the potent SERCA2a activating microprotein DWORF.^14^^,^^84^^,^^87^^,^^88^ DWORF is highly expressed in the heart and soleus, and it has been shown that DWORF activates SERCA2a activity in part by displacing the SERCA2a inhibitor PLN, leading to increased peak systolic Ca^2+^ transients, SR Ca^2+^ load, and SERCA2a enzymatic activity.^14^ DWORF protein and mRNA levels are decreased in various models of heart failure and DWORF overexpression in genetic^84^^,^^88^ and experimental^87^ mouse models of heart failure using transgenic or AAV-mediated gene delivery systems is cardioprotective and reduces disease pathogenesis. These studies highlight the potential to use microproteins as therapeutics to modulate the activity of ion transporters such as SERCA2a and active development of DWORF as a heart failure therapeutic is currently ongoing.^84^^,^^87^^,^^88^

#### MOTS-c

MOTS-c is a microprotein that is encoded in mitochondrial DNA (mtDNA), which was first demonstrated to target skeletal muscle and regulate insulin sensitivity and glucose metabolism.^15^^,^^129^ MOTS-c is detected in the circulation, indicating it is a mitochondrial hormone, or “mitokine”.^15^^,^^129^^,^^130^ Treatment of mice with MOTS-c increases insulin sensitivity under high fat diet conditions and confers resistance to obesity.^15^ MOTS-c has also been shown to relieve hyperglycemia and insulin resistance in gestational diabetes mellitus in a high fat diet/low-dose-streptozotocin mouse model^131^ and reduces lipid accumulation and fatty acid levels in livers of mice fed a normal diet.^132^ Additionally, there have been reports that circulating MOTS-c levels are reduced in obese male children and adolescents, and the decrease is further exacerbated in patients who also have insulin resistance.^133^ The mechanism of action of MOTS-c has been an active area of investigation and studies have found that it primarily acts on skeletal muscle and the heart, increases cellular levels of AICAR (an AMP-activated protein kinase [AMPK] agonist), activates AMPK, and helps maintain metabolic flexibility and homeostasis.^15^^,^^130^ MOTS-c has also been shown to translocate to the nucleus and affects gene expression during metabolic stress.^134^ MOTS-c has been labeled as an “exercise mimetic” as it has been shown to confer some exercise-like effects by lowering blood glucose levels and holds promise to treat obesity and type 2 diabetes.^15^^,^^130^ Additionally, another group has published interesting findings demonstrating that MOTS-c modulates the TGF-β/SMAD-signaling pathway and can improve osteoporosis.^135^^,^^136^

### Targeting microproteins in cancer

Numerous microproteins have been implicated in cancer as key players in cell proliferation, tumor suppression, invasion, and metastasis.^75^^,^^137^^,^^138^ Here we describe several candidate microproteins that represent potential therapeutic targets using overexpression (CIP2A-BP, MIAC, MP31) or inhibition (ASAP, APPLE) strategies (Figure 3).

#### CIP2A-BP

Encoded by the ncRNA LINC00665, the 52-AA microprotein CIP2A-BP was identified as being downregulated by TGF-β in breast cancer cell lines, and low levels of CIP2A-BP expression are associated with poor survival in triple-negative breast cancer patients.^139^ CIP2A-BP was shown to compete with protein phosphatase 2 (PP2A) to bind to CIP2A (cancerous inhibitor of PP2A, cancer inhibitory factor) and inhibit activation of the PI3K/AKT/NFkB pathway, preventing the migration and invasion of triple-negative breast cancer cells both *in vitro* and *in vivo.*^139^ Thus, CIP2A-BP represents a new microprotein target that can be overexpressed to treat triple-negative breast cancer metastasis.

#### MIAC

Microprotein inhibiting actin cytoskeleton (MIAC) was recently identified as a 51-AA microprotein encoded by the ncRNA RP11-469H8.6 that is significantly decreased in head and neck squamous cell carcinoma and renal cell carcinoma and is positively correlated with disease.^140^^,^^141^ MIAC was shown to directly interact with Aquaporin 2 and inhibit the actin cytoskeleton by regulating the Septin 2/Integrin Beta 4 pathway to suppress tumor growth and metastasis.^141^ Additionally, the tumor suppressor potential of MIAC was recently demonstrated in renal cell carcinoma where MIAC overexpression inhibited tumor growth of renal cancer cells in a subcutaneous xenograft mouse model by inhibiting epiregulin/epidermal growth factor receptor signaling.^140^

#### MP31

Extensive ribosome profiling analysis of human glioblastoma (GBM) samples and paired normal brain tissues identified MP31 as a 31-AA microprotein translated from the 5′ UTR of the well described tumor suppressor PTEN.^142^ Under normal conditions, MP31 binds to lactate dehydrogenase B (LDHB) to suppress lactate-pyruvate conversion, and downregulation of MP31 in GBM cells was associated with increased lactate utilization, a characteristic metabolic finding of cancer cells with high tumorigenicity.^143^ Patient-derived GBM cell lines exhibited downregulation of MP31, and MP31 overexpression in these cells led to reduced tumorigenicity. Additionally, systemic injection of recombinant MP31 reduced tumor size in mice in a patient-derived xenograft *in situ* GBM model leading to prolonged survival.^142^

#### ASAP

The 94-AA microprotein ASAP (ATP synthase-associated peptide) was identified as a highly upregulated gene (ncRNA LINC00467) in HCT116 colorectal cancer (CRC) cells and its expression predicts poor outcomes in CRC patients.^144^ ASAP was demonstrated to interact with ATP synthase and enhance mitochondrial ATP production, resulting in increased CRC cell proliferation *in vitro* and *in vivo*. Targeting ASAP in patient-derived xenografts using CRISPR/Cas9 resulted in the inhibition of CRC cell proliferation and reduced tumor size, suggesting that targeting ASAP in CRC may be therapeutically beneficial.^144^

#### APPLE

The 90-AA microprotein APPLE was identified from Ribo-seq data from hematopoietic malignancies^145^ and publicly available MS data.^41^ APPLE is highly expressed in acute myeloid leukemia (AML) patient samples and its expression is associated with poor outcomes in hematopoietic malignancies.^145^ Termed an “oncomicroprotein”, APPLE was shown to localize on the ribosome-bound ER and interact with translation elongation factors and poly(A)-binding proteins to promote mRNA looping and eIF4A complex assembly to regulate the translation of a subset of mRNAs that contribute to AML progression, thus supporting a pro-cancer translation program.^145^ Targeting APPLE using shRNA resulted in broad anti-cancer effects both *in vitro* and *in vivo*, indicating that targeting APPLE may be a clinically relevant approach to inhibit oncoprotein synthesis in cancer cells.^145^

## Conclusions and future directions

Since the recent genesis of the microprotein field, many sORF-encoded microproteins have been defined as key players in cellular physiology. However, it is hypothesized that there are still a significant number of microproteins hidden in the genome that have yet to be identified, validated, and functionally characterized. Numerous experimental methods, computational tools and bioinformatic platforms have been developed and applied to discover microproteins and these tools are currently undergoing further optimization to increase their sensitivity and reliability. In this review we have highlighted many of these powerful tools which incorporate sequencing- and MS-based discovery, domain prediction, and evolutionary conservation that have been used in isolation or in combination to robustly identify sORF-encoded microproteins. Following their identification and experimental validation, extensive functional characterization of candidate microproteins will help elucidate contributions to cellular and whole-body physiology. Additionally, in the future it may be advantageous to apply new artificial intelligence tools such as Alpha-Fold to predict microprotein structure from their AA sequences to give insight into their putative functions^146^ or develop new drugs to target microprotein activity.

As comprehensively demonstrated in this review, many microproteins that have been identified thus far localize to discrete subcellular domains where they regulate pivotal cellular processes, often via their direct interaction with larger proteins and/or multi-protein complexes. Due to their unique ability to endogenously regulate and fine-tune specific physiological processes, microproteins represent attractive potential therapeutic targets for future development. Herein, we have highlighted the ongoing discovery of microproteins in parallel with advances in bioinformatics, experimental methods, and therapeutic development that collectively contribute to the exciting future of microprotein biology.

## References

[1] Ruiz-Orera J., Messeguer X., Subirana J.A., Alba M.M. (2014). Long non-coding RNAs as a source of new peptides. Elife.

[2] Bazzini A.A., Johnstone T.G., Christiano R., Mackowiak S.D., Obermayer B., Fleming E.S., Vejnar C.E., Lee M.T., Rajewsky N., Walther T.C., Giraldez A.J. (2014). Identification of small ORFs in vertebrates using ribosome footprinting and evolutionary conservation. EMBO J..

[3] Mackowiak S.D., Zauber H., Bielow C., Thiel D., Kutz K., Calviello L., Mastrobuoni G., Rajewsky N., Kempa S., Selbach M., Obermayer B. (2015). Extensive identification and analysis of conserved small ORFs in animals. Genome Biol..

[4] Saghatelian A., Couso J.P. (2015). Discovery and characterization of smORF-encoded bioactive polypeptides. Nat. Chem. Biol..

[5] Martinez T.F., Chu Q., Donaldson C., Tan D., Shokhirev M.N., Saghatelian A. (2020). Accurate annotation of human protein-coding small open reading frames. Nat. Chem. Biol..

[6] Basrai M.A., Hieter P., Boeke J.D. (1997). Small open reading frames: beautiful needles in the haystack. Genome Res..

[7] Kitabchi A.E. (1977). Proinsulin and C-peptide: a review. Metabolism.

[8] Chang M.M., Leeman S.E., Niall H.D. (1971). Amino-acid sequence of substance P. Nat. New Biol..

[9] Wulff B.S., Johansen T.E., Dalbøge H., O'Hare M.M., Schwartz T.W. (1993). Processing of two homologous precursors, pro-neuropeptide Y and pro-pancreatic polypeptide, in transfected cell lines expressing different precursor convertases. J. Biol. Chem..

[10] Rouillé Y., Duguay S.J., Lund K., Furuta M., Gong Q., Lipkind G., Oliva A.A., Chan S.J., Steiner D.F. (1995). Proteolytic processing mechanisms in the biosynthesis of neuroendocrine peptides: the subtilisin-like proprotein convertases. Front. Neuroendocrinol..

[11] Anderson D.M., Anderson K.M., Chang C.L., Makarewich C.A., Nelson B.R., McAnally J.R., Kasaragod P., Shelton J.M., Liou J., Bassel-Duby R., Olson E.N. (2015). A micropeptide encoded by a putative long noncoding RNA regulates muscle performance. Cell.

[12] Anderson D.M., Makarewich C.A., Anderson K.M., Shelton J.M., Bezprozvannaya S., Bassel-Duby R., Olson E.N. (2016). Widespread control of calcium signaling by a family of SERCA-inhibiting micropeptides. Sci. Signal..

[13] Makarewich C.A., Baskin K.K., Munir A.Z., Bezprozvannaya S., Sharma G., Khemtong C., Shah A.M., McAnally J.R., Malloy C.R., Szweda L.I. (2018). MOXI is a mitochondrial micropeptide that enhances fatty acid beta-oxidation. Cell Rep..

[14] Nelson B.R., Makarewich C.A., Anderson D.M., Winders B.R., Troupes C.D., Wu F., Reese A.L., McAnally J.R., Chen X., Kavalali E.T. (2016). A peptide encoded by a transcript annotated as long noncoding RNA enhances SERCA activity in muscle. Science.

[15] Lee C., Zeng J., Drew B.G., Sallam T., Martin-Montalvo A., Wan J., Kim S.J., Mehta H., Hevener A.L., de Cabo R., Cohen P. (2015). The mitochondrial-derived peptide MOTS-c promotes metabolic homeostasis and reduces obesity and insulin resistance. Cell Metabol..

[16] Ho L., Tan S.Y.X., Wee S., Wu Y., Tan S.J.C., Ramakrishna N.B., Chng S.C., Nama S., Szczerbinska I., Chan Y.S. (2015). ELABELA is an endogenous growth factor that sustains hESC self-renewal via the PI3K/AKT pathway. Cell Stem Cell.

[17] Starck S.R., Tsai J.C., Chen K., Shodiya M., Wang L., Yahiro K., Martins-Green M., Shastri N., Walter P. (2016). Translation from the 5' untranslated region shapes the integrated stress response. Science.

[18] Geering K. (2006). FXYD proteins: new regulators of Na-K-ATPase. Am. J. Physiol. Ren. Physiol..

[19] Cheung J.Y., Zhang X.Q., Song J., Gao E., Chan T.O., Rabinowitz J.E., Koch W.J., Feldman A.M., Wang J. (2013). Coordinated regulation of cardiac Na(+)/Ca (2+) exchanger and Na (+)-K (+)-ATPase by phospholemman (FXYD1). Adv. Exp. Med. Biol..

[20] Rathod N., Bak J.J., Primeau J.O., Fisher M.E., Espinoza-Fonseca L.M., Lemieux M.J., Young H.S. (2021). Nothing regular about the regulins: distinct functional properties of SERCA transmembrane peptide regulatory subunits. Int. J. Mol. Sci..

[21] Koss K.L., Kranias E.G. (1996). Phospholamban: a prominent regulator of myocardial contractility. Circ. Res..

[22] MacLennan D.H., Kranias E.G. (2003). Phospholamban: a crucial regulator of cardiac contractility. Nat. Rev. Mol. Cell Biol..

[23] Minamisawa S., Wang Y., Chen J., Ishikawa Y., Chien K.R., Matsuoka R. (2003). Atrial chamber-specific expression of sarcolipin is regulated during development and hypertrophic remodeling. J. Biol. Chem..

[24] Mistry J., Chuguransky S., Williams L., Qureshi M., Salazar G.A., Sonnhammer E.L.L., Tosatto S.C.E., Paladin L., Raj S., Richardson L.J. (2021). Pfam: the protein families database in 2021. Nucleic Acids Res..

[25] Lu S., Wang J., Chitsaz F., Derbyshire M.K., Geer R.C., Gonzales N.R., Gwadz M., Hurwitz D.I., Marchler G.H., Song J.S. (2020). CDD/SPARCLE: the conserved domain database in 2020. Nucleic Acids Res..

[26] Sonnhammer E.L., von Heijne G., Krogh A. (1998). A hidden Markov model for predicting transmembrane helices in protein sequences. Proc. Int. Conf. Intell. Syst. Mol. Biol..

[27] Schlesinger D., Elsässer S.J. (2022). Revisiting sORFs: overcoming challenges to identify and characterize functional microproteins. FEBS J..

[28] Wright B.W., Yi Z., Weissman J.S., Chen J. (2022). The dark proteome: translation from noncanonical open reading frames. Trends Cell Biol..

[29] Leong A.Z.X., Lee P.Y., Mohtar M.A., Syafruddin S.E., Pung Y.F., Low T.Y. (2022). Short open reading frames (sORFs) and microproteins: an update on their identification and validation measures. J. Biomed. Sci..

[30] Ingolia N.T., Ghaemmaghami S., Newman J.R.S., Weissman J.S. (2009). Genome-wide analysis in vivo of translation with nucleotide resolution using ribosome profiling. Science.

[31] Brar G.A., Weissman J.S. (2015). Ribosome profiling reveals the what, when, where and how of protein synthesis. Nat. Rev. Mol. Cell Biol..

[32] Guttman M., Russell P., Ingolia N.T., Weissman J.S., Lander E.S. (2013). Ribosome profiling provides evidence that large noncoding RNAs do not encode proteins. Cell.

[33] VanInsberghe M., van den Berg J., Andersson-Rolf A., Clevers H., van Oudenaarden A. (2021). Single-cell Ribo-seq reveals cell cycle-dependent translational pausing. Nature.

[34] He C., Jia C., Zhang Y., Xu P. (2018). Enrichment-based proteogenomics identifies microproteins, missing proteins, and novel smORFs in Saccharomyces cerevisiae. J. Proteome Res..

[35] Cao X., Khitun A., Luo Y., Na Z., Phoodokmai T., Sappakhaw K., Olatunji E., Uttamapinant C., Slavoff S.A. (2021). Alt-RPL36 downregulates the PI3K-AKT-mTOR signaling pathway by interacting with TMEM24. Nat. Commun..

[36] Ma J., Diedrich J.K., Jungreis I., Donaldson C., Vaughan J., Kellis M., Yates J.R., Saghatelian A. (2016). Improved identification and analysis of small open reading frame encoded polypeptides. Anal. Chem..

[37] Yang Y., Wang H., Zhang Y., Chen L., Chen G., Bao Z., Yang Y., Xie Z., Zhao Q. (2023). An optimized proteomics approach reveals novel alternative proteins in mouse liver development. Mol. Cell. Proteomics.

[38] Fijalkowski I., Peeters M.K.R., Van Damme P. (2021). Small protein enrichment improves proteomics detection of sORF encoded polypeptides. Front. Genet..

[39] Wang B., Wang Z., Pan N., Huang J., Wan C. (2021). Improved identification of small open reading frames encoded peptides by top-down proteomic approaches and de novo sequencing. Int. J. Mol. Sci..

[40] Pan N., Wang Z., Wang B., Wan J., Wan C. (2021). Mapping microproteins and ncRNA-encoded polypeptides in different mouse tissues. Front. Cell Dev. Biol..

[41] Slavoff S.A., Mitchell A.J., Schwaid A.G., Cabili M.N., Ma J., Levin J.Z., Karger A.D., Budnik B.A., Rinn J.L., Saghatelian A. (2013). Peptidomic discovery of short open reading frame-encoded peptides in human cells. Nat. Chem. Biol..

[42] Zhu Y., Orre L.M., Johansson H.J., Huss M., Boekel J., Vesterlund M., Fernandez-Woodbridge A., Branca R.M.M., Lehtiö J. (2018). Discovery of coding regions in the human genome by integrated proteogenomics analysis workflow. Nat. Commun..

[43] Bruderer R., Bernhardt O.M., Gandhi T., Xuan Y., Sondermann J., Schmidt M., Gomez-Varela D., Reiter L. (2017). Optimization of experimental parameters in data-independent mass spectrometry significantly increases depth and reproducibility of results. Mol. Cell. Proteomics.

[44] Na Z., Dai X., Zheng S.J., Bryant C.J., Loh K.H., Su H., Luo Y., Buhagiar A.F., Cao X., Baserga S.J. (2022). Mapping subcellular localizations of unannotated microproteins and alternative proteins with MicroID. Mol. Cell.

[45] Chu Q., Rathore A., Diedrich J.K., Donaldson C.J., Yates J.R., Saghatelian A. (2017). Identification of microprotein-protein interactions via APEX tagging. Biochemistry.

[46] Roux K.J., Kim D.I., Burke B., May D.G. (2018). BioID: a screen for protein-protein interactions. Curr. Protoc. Protein Sci..

[47] Hao Y., Zhang L., Niu Y., Cai T., Luo J., He S., Zhang B., Zhang D., Qin Y., Yang F., Chen R. (2018). SmProt: a database of small proteins encoded by annotated coding and non-coding RNA loci. Briefings Bioinf..

[48] Olexiouk V., Van Criekinge W., Menschaert G. (2018). An update on sORFs.org: a repository of small ORFs identified by ribosome profiling. Nucleic Acids Res..

[49] Mudge J.M., Ruiz-Orera J., Prensner J.R., Brunet M.A., Calvet F., Jungreis I., Gonzalez J.M., Magrane M., Martinez T.F., Schulz J.F. (2022). Standardized annotation of translated open reading frames. Nat. Biotechnol..

[50] Lin M.F., Jungreis I., Kellis M. (2011). PhyloCSF: a comparative genomics method to distinguish protein coding and non-coding regions. Bioinformatics.

[51] Zhu M., Gribskov M. (2019). MiPepid: MicroPeptide identification tool using machine learning. BMC Bioinf..

[52] Ji X., Cui C., Cui Q. (2020). smORFunction: a tool for predicting functions of small open reading frames and microproteins. BMC Bioinf..

[53] Skarshewski A., Stanton-Cook M., Huber T., Al Mansoori S., Smith R., Beatson S.A., Rothnagel J.A. (2014). uPEPperoni: an online tool for upstream open reading frame location and analysis of transcript conservation. BMC Bioinf..

[54] Brunet M.A., Lucier J.F., Levesque M., Leblanc S., Jacques J.F., Al-Saedi H.R.H., Guilloy N., Grenier F., Avino M., Fournier I. (2021). OpenProt 2021: deeper functional annotation of the coding potential of eukaryotic genomes. Nucleic Acids Res..

[55] Zhou P., Silverstein K.A., Gao L., Walton J.D., Nallu S., Guhlin J., Young N.D. (2013). Detecting small plant peptides using SPADA (small peptide alignment discovery application). BMC Bioinf..

[56] Makarewich C.A., Munir A.Z., Bezprozvannaya S., Gibson A.M., Young Kim S., Martin-Sandoval M.S., Mathews T.P., Szweda L.I., Bassel-Duby R., Olson E.N. (2022). The cardiac-enriched microprotein mitolamban regulates mitochondrial respiratory complex assembly and function in mice. Proc. Natl. Acad. Sci. USA.

[57] Chu Q., Martinez T.F., Novak S.W., Donaldson C.J., Tan D., Vaughan J.M., Chang T., Diedrich J.K., Andrade L., Kim A. (2019). Regulation of the ER stress response by a mitochondrial microprotein. Nat. Commun..

[58] Senís E., Esgleas M., Najas S., Jiménez-Sábado V., Bertani C., Giménez-Alejandre M., Escriche A., Ruiz-Orera J., Hergueta-Redondo M., Jiménez M. (2021). TUNAR lncRNA encodes a microprotein that regulates neural differentiation and neurite formation by modulating calcium dynamics. Front. Cell Dev. Biol..

[59] Boix O., Martinez M., Vidal S., Giménez-Alejandre M., Palenzuela L., Lorenzo-Sanz L., Quevedo L., Moscoso O., Ruiz-Orera J., Ximénez-Embún P. (2022). pTINCR microprotein promotes epithelial differentiation and suppresses tumor growth through CDC42 SUMOylation and activation. Nat. Commun..

[60] Brito-Estrada O., Hassel K.R., Makarewich C.A. (2022). An integrated approach for microprotein identification and sequence analysis. J. Vis. Exp..

[61] Makarewich C.A., Olson E.N. (2017). Mining for micropeptides. Trends Cell Biol..

[62] Sweadner K.J., Rael E. (2000). The FXYD gene family of small ion transport regulators or channels: cDNA sequence, protein signature sequence, and expression. Genomics.

[63] Therien A.G., Goldshleger R., Karlish S.J., Blostein R. (1997). Tissue-specific distribution and modulatory role of the gamma subunit of the Na,K-ATPase. J. Biol. Chem..

[64] Meij I.C., Koenderink J.B., van Bokhoven H., Assink K.F., Groenestege W.T., de Pont J.J., Bindels R.J., Monnens L.A., van den Heuvel L.P., Knoers N.V. (2000). Dominant isolated renal magnesium loss is caused by misrouting of the Na(+),K(+)-ATPase gamma-subunit. Nat. Genet..

[65] Bogaev R.C., Jia L.G., Kobayashi Y.M., Palmer C.J., Mounsey J.P., Moorman J.R., Jones L.R., Tucker A.L. (2001). Gene structure and expression of phospholemman in mouse. Gene.

[66] Wang J., Zhang X.Q., Ahlers B.A., Carl L.L., Song J., Rothblum L.I., Stahl R.C., Carey D.J., Cheung J.Y. (2006). Cytoplasmic tail of phospholemman interacts with the intracellular loop of the cardiac Na+/Ca2+ exchanger. J. Biol. Chem..

[67] Fuller W., Tulloch L.B., Shattock M.J., Calaghan S.C., Howie J., Wypijewski K.J. (2013). Regulation of the cardiac sodium pump. Cell. Mol. Life Sci..

[68] Ahlers B.A., Zhang X.Q., Moorman J.R., Rothblum L.I., Carl L.L., Song J., Wang J., Geddis L.M., Tucker A.L., Mounsey J.P., Cheung J.Y. (2005). Identification of an endogenous inhibitor of the cardiac Na+/Ca2+ exchanger, phospholemman. J. Biol. Chem..

[69] Fu H., Wang T., Kong X., Yan K., Yang Y., Cao J., Yuan Y., Wang N., Kee K., Lu Z.J., Xi Q. (2022). A Nodal enhanced micropeptide NEMEP regulates glucose uptake during mesendoderm differentiation of embryonic stem cells. Nat. Commun..

[70] Quinn M.E., Goh Q., Kurosaka M., Gamage D.G., Petrany M.J., Prasad V., Millay D.P. (2017). Myomerger induces fusion of non-fusogenic cells and is required for skeletal muscle development. Nat. Commun..

[71] Bi P., Ramirez-Martinez A., Li H., Cannavino J., McAnally J.R., Shelton J.M., Sánchez-Ortiz E., Bassel-Duby R., Olson E.N. (2017). Control of muscle formation by the fusogenic micropeptide myomixer. Science.

[72] Zhang Q., Vashisht A.A., O'Rourke J., Corbel S.Y., Moran R., Romero A., Miraglia L., Zhang J., Durrant E., Schmedt C. (2017). The microprotein Minion controls cell fusion and muscle formation. Nat. Commun..

[73] Leikina E., Gamage D.G., Prasad V., Goykhberg J., Crowe M., Diao J., Kozlov M.M., Chernomordik L.V., Millay D.P. (2018). Myomaker and myomerger work independently to control distinct steps of membrane remodeling during myoblast fusion. Dev. Cell.

[74] Yang C., Wang X. (2021). Lysosome biogenesis: regulation and functions. J. Cell Biol..

[75] Polycarpou-Schwarz M., Groß M., Mestdagh P., Schott J., Grund S.E., Hildenbrand C., Rom J., Aulmann S., Sinn H.P., Vandesompele J., Diederichs S. (2018). The cancer-associated microprotein CASIMO1 controls cell proliferation and interacts with squalene epoxidase modulating lipid droplet formation. Oncogene.

[76] Matsumoto A., Clohessy J.G., Pandolfi P.P. (2017). SPAR, a lncRNA encoded mTORC1 inhibitor. Cell Cycle.

[77] Matsumoto A., Pasut A., Matsumoto M., Yamashita R., Fung J., Monteleone E., Saghatelian A., Nakayama K.I., Clohessy J.G., Pandolfi P.P. (2017). mTORC1 and muscle regeneration are regulated by the LINC00961-encoded SPAR polypeptide. Nature.

[78] Zhang P., Liang X., Shan T., Jiang Q., Deng C., Zheng R., Kuang S. (2015). mTOR is necessary for proper satellite cell activity and skeletal muscle regeneration. Biochem. Biophys. Res. Commun..

[79] Pueyo J.I., Magny E.G., Sampson C.J., Amin U., Evans I.R., Bishop S.A., Couso J.P. (2016). Hemotin, a regulator of phagocytosis encoded by a small ORF and conserved across metazoans. PLoS Biol..

[80] Clapham D.E. (2007). Calcium signaling. Cell.

[81] Periasamy M., Kalyanasundaram A. (2007). SERCA pump isoforms: their role in calcium transport and disease. Muscle Nerve.

[82] Bers D.M. (2002). Cardiac excitation-contraction coupling. Nature.

[83] Luo W., Grupp I.L., Harrer J., Ponniah S., Grupp G., Duffy J.J., Doetschman T., Kranias E.G. (1994). Targeted ablation of the phospholamban gene is associated with markedly enhanced myocardial contractility and loss of beta-agonist stimulation. Circ. Res..

[84] Makarewich C.A., Munir A.Z., Schiattarella G.G., Bezprozvannaya S., Raguimova O.N., Cho E.E., Vidal A.H., Robia S.L., Bassel-Duby R., Olson E.N. (2018). The DWORF micropeptide enhances contractility and prevents heart failure in a mouse model of dilated cardiomyopathy. Elife.

[85] Fisher M.E., Bovo E., Aguayo-Ortiz R., Cho E.E., Pribadi M.P., Dalton M.P., Rathod N., Lemieux M.J., Espinoza-Fonseca L.M., Robia S.L. (2021). Dwarf open reading frame (DWORF) is a direct activator of the sarcoplasmic reticulum calcium pump SERCA. Elife.

[86] Li A., Yuen S.L., Stroik D.R., Kleinboehl E., Cornea R.L., Thomas D.D. (2021). The transmembrane peptide DWORF activates SERCA2a via dual mechanisms. J. Biol. Chem..

[87] Makarewich C.A., Bezprozvannaya S., Gibson A.M., Bassel-Duby R., Olson E.N. (2020). Gene therapy with the DWORF micropeptide attenuates cardiomyopathy in mice. Circ. Res..

[88] Morales E.D., Yue Y., Watkins T.B., Han J., Pan X., Gibson A.M., Hu B., Brito-Estrada O., Yao G., Makarewich C.A. (2023). Dwarf open reading frame (DWORF) gene therapy ameliorated duchenne muscular dystrophy cardiomyopathy in aged mdx mice. J. Am. Heart Assoc..

[89] Zhang S., Reljić B., Liang C., Kerouanton B., Francisco J.C., Peh J.H., Mary C., Jagannathan N.S., Olexiouk V., Tang C. (2020). Mitochondrial peptide BRAWNIN is essential for vertebrate respiratory complex III assembly. Nat. Commun..

[90] van Heesch S., Witte F., Schneider-Lunitz V., Schulz J.F., Adami E., Faber A.B., Kirchner M., Maatz H., Blachut S., Sandmann C.L. (2019). The translational landscape of the human heart. Cell.

[91] Stein C.S., Jadiya P., Zhang X., McLendon J.M., Abouassaly G.M., Witmer N.H., Anderson E.J., Elrod J.W., Boudreau R.L. (2018). Mitoregulin: a lncRNA-encoded microprotein that supports mitochondrial supercomplexes and respiratory efficiency. Cell Rep..

[92] Chugunova A., Loseva E., Mazin P., Mitina A., Navalayeu T., Bilan D., Vishnyakova P., Marey M., Golovina A., Serebryakova M. (2019). LINC00116 codes for a mitochondrial peptide linking respiration and lipid metabolism. Proc. Natl. Acad. Sci. USA.

[93] Friesen M., Warren C.R., Yu H., Toyohara T., Ding Q., Florido M.H.C., Sayre C., Pope B.D., Goff L.A., Rinn J.L., Cowan C.A. (2020). Mitoregulin controls beta-oxidation in human and mouse adipocytes. Stem Cell Rep..

[94] Averina O.A., Permyakov O.A., Emelianova M.A., Grigoryeva O.O., Gulyaev M.V., Pavlova O.S., Mariasina S.S., Frolova O.Y., Kurkina M.V., Baydakova G.V. (2023). Mitochondrial peptide Mtln contributes to oxidative metabolism in mice. Biochimie.

[95] Liang C., Zhang S., Robinson D., Ploeg M.V., Wilson R., Nah J., Taylor D., Beh S., Lim R., Sun L. (2022). Mitochondrial microproteins link metabolic cues to respiratory chain biogenesis. Cell Rep..

[96] Desmurs M., Foti M., Raemy E., Vaz F.M., Martinou J.C., Bairoch A., Lane L. (2015). C11orf83, a mitochondrial cardiolipin-binding protein involved in bc1 complex assembly and supercomplex stabilization. Mol. Cell Biol..

[97] Dennerlein S., Poerschke S., Oeljeklaus S., Wang C., Richter-Dennerlein R., Sattmann J., Bauermeister D., Hanitsch E., Stoldt S., Langer T. (2021). Defining the interactome of the human mitochondrial ribosome identifies SMIM4 and TMEM223 as respiratory chain assembly factors. Elife.

[98] Shpilka T., Haynes C.M. (2018). The mitochondrial UPR: mechanisms, physiological functions and implications in ageing. Nat. Rev. Mol. Cell Biol..

[99] Scheper W., Hoozemans J.J.M. (2015). The unfolded protein response in neurodegenerative diseases: a neuropathological perspective. Acta Neuropathol..

[100] Kang M., Tang B., Li J., Zhou Z., Liu K., Wang R., Jiang Z., Bi F., Patrick D., Kim D. (2020). Identification of miPEP133 as a novel tumor-suppressor microprotein encoded by miR-34a pri-miRNA. Mol. Cancer.

[101] Qiao P., Li G., Bi W., Yang L., Yao L., Wu D. (2015). microRNA-34a inhibits epithelial mesenchymal transition in human cholangiocarcinoma by targeting Smad4 through transforming growth factor-beta/Smad pathway. BMC Cancer.

[102] Tazawa H., Tsuchiya N., Izumiya M., Nakagama H. (2007). Tumor-suppressive miR-34a induces senescence-like growth arrest through modulation of the E2F pathway in human colon cancer cells. Proc. Natl. Acad. Sci. USA.

[103] Wang X., Li J., Dong K., Lin F., Long M., Ouyang Y., Wei J., Chen X., Weng Y., He T., Zhang H. (2015). Tumor suppressor miR-34a targets PD-L1 and functions as a potential immunotherapeutic target in acute myeloid leukemia. Cell. Signal..

[104] Hashimoto Y., Niikura T., Tajima H., Yasukawa T., Sudo H., Ito Y., Kita Y., Kawasumi M., Kouyama K., Doyu M. (2001). A rescue factor abolishing neuronal cell death by a wide spectrum of familial Alzheimer's disease genes and Abeta. Proc. Natl. Acad. Sci. USA.

[105] Guo B., Zhai D., Cabezas E., Welsh K., Nouraini S., Satterthwait A.C., Reed J.C. (2003). Humanin peptide suppresses apoptosis by interfering with Bax activation. Nature.

[106] Hashimoto Y., Kurita M., Aiso S., Nishimoto I., Matsuoka M. (2009). Humanin inhibits neuronal cell death by interacting with a cytokine receptor complex or complexes involving CNTF receptor alpha/WSX-1/gp130. Mol. Biol. Cell.

[107] Ying G., Iribarren P., Zhou Y., Gong W., Zhang N., Yu Z.X., Le Y., Cui Y., Wang J.M. (2004). Humanin, a newly identified neuroprotective factor, uses the G protein-coupled formylpeptide receptor-like-1 as a functional receptor. J. Immunol..

[108] Thiankhaw K., Chattipakorn K., Chattipakorn S.C., Chattipakorn N. (2022). Roles of humanin and derivatives on the pathology of neurodegenerative diseases and cognition. Biochim. Biophys. Acta Gen. Subj..

[109] Boutari C., Pappas P.D., Theodoridis T.D., Vavilis D. (2022). Humanin and diabetes mellitus: a review of in vitro and in vivo studies. World J. Diabetes.

[110] Cai H., Liu Y., Men H., Zheng Y. (2021). Protective mechanism of humanin against oxidative stress in aging-related cardiovascular diseases. Front. Endocrinol..

[111] D'Lima N.G., Ma J., Winkler L., Chu Q., Loh K.H., Corpuz E.O., Budnik B.A., Lykke-Andersen J., Saghatelian A., Slavoff S.A. (2017). A human microprotein that interacts with the mRNA decapping complex. Nat. Chem. Biol..

[112] Luo Y., Na Z., Slavoff S.A. (2018). P-bodies: composition, properties, and functions. Biochemistry.

[113] Agarwal S., Harada J., Schreifels J., Lech P., Nikolai B., Yamaguchi T., Chanda S.K., Somia N.V. (2006). Isolation, characterization, and genetic complementation of a cellular mutant resistant to retroviral infection. Proc. Natl. Acad. Sci. USA.

[114] Arnoult N., Correia A., Ma J., Merlo A., Garcia-Gomez S., Maric M., Tognetti M., Benner C.W., Boulton S.J., Saghatelian A., Karlseder J. (2017). Regulation of DNA repair pathway choice in S and G2 phases by the NHEJ inhibitor CYREN. Nature.

[115] Slavoff S.A., Heo J., Budnik B.A., Hanakahi L.A., Saghatelian A. (2014). A human short open reading frame (sORF)-encoded polypeptide that stimulates DNA end joining. J. Biol. Chem..

[116] Kretz M., Siprashvili Z., Chu C., Webster D.E., Zehnder A., Qu K., Lee C.S., Flockhart R.J., Groff A.F., Chow J. (2013). Control of somatic tissue differentiation by the long non-coding RNA TINCR. Nature.

[117] Daya S., Berns K.I. (2008). Gene therapy using adeno-associated virus vectors. Clin. Microbiol. Rev..

[118] Grieger J.C., Samulski R.J. (2005). Packaging capacity of adeno-associated virus serotypes: impact of larger genomes on infectivity and postentry steps. J. Virol..

[119] Chen J., Guo Z., Tian H., Chen X. (2016). Production and clinical development of nanoparticles for gene delivery. Mol. Ther. Methods Clin. Dev..

[120] Roberts T.C., Langer R., Wood M.J.A. (2020). Advances in oligonucleotide drug delivery. Nat. Rev. Drug Discov..

[121] Piacentino V., Weber C.R., Chen X., Weisser-Thomas J., Margulies K.B., Bers D.M., Houser S.R. (2003). Cellular basis of abnormal calcium transients of failing human ventricular myocytes. Circ. Res..

[122] del Monte F., Williams E., Lebeche D., Schmidt U., Rosenzweig A., Gwathmey J.K., Lewandowski E.D., Hajjar R.J. (2001). Improvement in survival and cardiac metabolism after gene transfer of sarcoplasmic reticulum Ca(2+)-ATPase in a rat model of heart failure. Circulation.

[123] Luo M., Anderson M.E. (2013). Mechanisms of altered Ca(2)(+) handling in heart failure. Circ. Res..

[124] Gwathmey J.K., Yerevanian A., Hajjar R.J. (2013). Targeting sarcoplasmic reticulum calcium ATPase by gene therapy. Hum. Gene Ther..

[125] Penny W.F., Hammond H.K. (2017). Randomized clinical trials of gene transfer for heart failure with reduced ejection fraction. Hum. Gene Ther..

[126] Wasala N.B., Yue Y., Lostal W., Wasala L.P., Niranjan N., Hajjar R.J., Babu G.J., Duan D. (2020). Single SERCA2a therapy ameliorated dilated cardiomyopathy for 18 Months in a mouse model of duchenne muscular dystrophy. Mol. Ther..

[127] Jessup M., Greenberg B., Mancini D., Cappola T., Pauly D.F., Jaski B., Yaroshinsky A., Zsebo K.M., Dittrich H., Hajjar R.J., Calcium Upregulation by Percutaneous Administration of Gene Therapy in Cardiac Disease CUPID Investigators (2011). Calcium upregulation by percutaneous administration of gene therapy in cardiac disease (CUPID): a phase 2 trial of intracoronary gene therapy of sarcoplasmic reticulum Ca2+-ATPase in patients with advanced heart failure. Circulation.

[128] Cutler M.J., Wan X., Plummer B.N., Liu H., Deschenes I., Laurita K.R., Hajjar R.J., Rosenbaum D.S. (2012). Targeted sarcoplasmic reticulum Ca2+ ATPase 2a gene delivery to restore electrical stability in the failing heart. Circulation.

[129] Zarse K., Ristow M. (2015). A mitochondrially encoded hormone ameliorates obesity and insulin resistance. Cell Metabol..

[130] Lee C., Kim K.H., Cohen P. (2016). MOTS-c: a novel mitochondrial-derived peptide regulating muscle and fat metabolism. Free Radic. Biol. Med..

[131] Yin Y., Pan Y., He J., Zhong H., Wu Y., Ji C., Liu L., Cui X. (2022). The mitochondrial-derived peptide MOTS-c relieves hyperglycemia and insulin resistance in gestational diabetes mellitus. Pharmacol. Res..

[132] Li Q., Lu H., Hu G., Ye Z., Zhai D., Yan Z., Wang L., Xiang A., Lu Z. (2019). Earlier changes in mice after D-galactose treatment were improved by mitochondria derived small peptide MOTS-c. Biochem. Biophys. Res. Commun..

[133] Du C., Zhang C., Wu W., Liang Y., Wang A., Wu S., Zhao Y., Hou L., Ning Q., Luo X. (2018). Circulating MOTS-c levels are decreased in obese male children and adolescents and associated with insulin resistance. Pediatr. Diabetes.

[134] Kim K.H., Son J.M., Benayoun B.A., Lee C. (2018). The mitochondrial-encoded peptide MOTS-c translocates to the nucleus to regulate nuclear gene expression in response to metabolic stress. Cell Metabol..

[135] Che N., Qiu W., Wang J.K., Sun X.X., Xu L.X., Liu R., Gu L. (2019). MOTS-c improves osteoporosis by promoting the synthesis of type I collagen in osteoblasts via TGF-beta/SMAD signaling pathway. Eur. Rev. Med. Pharmacol. Sci..

[136] Hu B.T., Chen W.Z. (2018). MOTS-c improves osteoporosis by promoting osteogenic differentiation of bone marrow mesenchymal stem cells via TGF-beta/Smad pathway. Eur. Rev. Med. Pharmacol. Sci..

[137] Merino-Valverde I., Greco E., Abad M. (2020). The microproteome of cancer: from invisibility to relevance. Exp. Cell Res..

[138] Liu Y., Zeng S., Wu M. (2022). Novel insights into noncanonical open reading frames in cancer. Biochim. Biophys. Acta Rev. Canc.

[139] Guo B., Wu S., Zhu X., Zhang L., Deng J., Li F., Wang Y., Zhang S., Wu R., Lu J., Zhou Y. (2020). Micropeptide CIP2A-BP encoded by LINC00665 inhibits triple-negative breast cancer progression. EMBO J..

[140] Li M., Liu G., Jin X., Guo H., Setrerrahmane S., Xu X., Li T., Lin Y., Xu H. (2022). Micropeptide MIAC inhibits the tumor progression by interacting with AQP2 and inhibiting EREG/EGFR signaling in renal cell carcinoma. Mol. Cancer.

[141] Li M., Li X., Zhang Y., Wu H., Zhou H., Ding X., Zhang X., Jin X., Wang Y., Yin X. (2020). Micropeptide MIAC inhibits HNSCC progression by interacting with Aquaporin 2. J. Am. Chem. Soc..

[142] Huang N., Li F., Zhang M., Zhou H., Chen Z., Ma X., Yang L., Wu X., Zhong J., Xiao F. (2021). An upstream open reading frame in phosphatase and tensin homolog encodes a circuit breaker of lactate metabolism. Cell Metabol..

[143] Chen J., McKay R.M., Parada L.F. (2012). Malignant glioma: lessons from genomics, mouse models, and stem cells. Cell.

[144] Ge Q., Jia D., Cen D., Qi Y., Shi C., Li J., Sang L., Yang L.J., He J., Lin A. (2021). Micropeptide ASAP encoded by LINC00467 promotes colorectal cancer progression by directly modulating ATP synthase activity. J. Clin. Invest..

[145] Sun L., Wang W., Han C., Huang W., Sun Y., Fang K., Zeng Z., Yang Q., Pan Q., Chen T. (2021). The oncomicropeptide APPLE promotes hematopoietic malignancy by enhancing translation initiation. Mol. Cell.

[146] Senior A.W., Evans R., Jumper J., Kirkpatrick J., Sifre L., Green T., Qin C., Žídek A., Nelson A.W.R., Bridgland A. (2019). Protein structure prediction using multiple deep neural networks in the 13th Critical Assessment of Protein Structure Prediction (CASP13). Proteins.

